# Morphological and Clinical Patterns of Paederus Dermatitis

**DOI:** 10.7759/cureus.58813

**Published:** 2024-04-23

**Authors:** Annie Priya Dharshini Inbamani, Gayathri Sundaram, Rajalakshmi Ramalingam

**Affiliations:** 1 Dermatology, Srinivasan Medical College and Hospital, Trichy, IND

**Keywords:** dermatitis, beetles, erythematous vesicular lesions, india, paederus dermatitis

## Abstract

Background

The objective of the present study was to describe the morphological and clinical patterns of paederus dermatitis (PD).

Methodology

This retrospective case series was conducted in the outpatient department of the Department of Dermatology, Srinivasan Medical College and Hospital, Trichy, Tamil Nadu, between June 2023 and August 2023 among patients with a clinical diagnosis of PD.

Results

This study included a total of 10 patients. The mean (SD) age of the patients was 19.4 (1.9) years. More than half of the patients (60.0%) were males. Of the 10 patients included, four (40.0%) were from rural areas, three (30.0%) were from urban areas, and three (30.0%) were from semi-urban areas. The maximum number of cases was reported between June and September. The most common presenting complaint was a burning sensation in 80.0% of the patients, followed by pain in 80.0% and blisters in 20.0% of the patients. The mean (SD) duration of the lesion was 4.2 (1.3) days. Regarding the clinical pattern of lesions, linear lesions were the most common (40.0%), followed by erythematous lesions with central gray area in 30.0%, kissing lesions in 20.0%, and burnt appearance in 10.0% of the lesions. Nearly half of the patients presented with lesions in the face (40.0%), the most common site in the present study, followed by lesions in the leg (20.0%), and lesions in the axilla, chest, arm, and back (10.0% each).

Conclusions

Understanding the epidemiology and clinical manifestations of this condition is crucial for accurate diagnosis, timely management, and public health interventions aimed at preventing *Paederus* beetle-related dermatitis.

## Introduction

The knowledge of beetles causing skin blisters dates back to ancient times, as documented by Castellani and Chalmers (1919), who noted this during the era of Archigenes, a contemporary of Celsus [1]. Patton (1929) mentioned approximately 250 known species of *Paederus* beetles, most of which produce vesicant fluid [2]. Despite their similarities in appearance, *Paederus* beetles exhibit slight variations in color, shape, and thoracic structure, which are discernible under appropriate magnification. Various species of Paederus beetles, including *Paederus brasilensis* (referred to as Podo), *P. colombius*, *P. fusipes*, and *P. peregrinus*, are responsible for causing paederus dermatitis (PD) in regions such as Venezuela, Indonesia, South America, and Taiwan [3,4].

Cameron (1931) documented around 43 species of *Paederus* beetles in the Indian peninsula [5]. Beetles are attracted to neon and fluorescent light sources [6]. PD serves as an entomological model for irritant contact dermatitis. In India, PD is commonly caused by *P. melampus*. PD is caused by paederin, the toxin produced by endosymbiotic *Pseudomonas* bacteria in the hemolymph and released by the female *Paederus* beetle. The most common groups of beetle-causing dermatitis in humans belong to the order Coleoptera, genus *Paederus*, vernacularly known as “Acid puchi”), family Oedemeridae, Meloidae, and Staphylinidae. *Paederus* beetle (also called Rove beetles) is a subtype of Staphylinidae. The irritant chemical present in the body fluids of these insects induces acute irritant contact dermatitis, characterized by erythematous vesicular lesions accompanied by a burning sensation on exposed skin areas. Against this background, this study aimed to describe the morphological and clinical patterns of PD using a retrospective case series.

## Materials and methods

This was a retrospective case series conducted in the outpatient department (OPD) of the Department of Dermatology, Srinivasan Medical College and Hospital, Trichy, Tamil Nadu, between June 2023 and August 2023. The study received approval from the Institute Human Ethics Committee. Participants were given the Participant Information Sheet in their local language and had the contents explained to them thoroughly in their language until they were satisfied. Enrolment in the study occurred only after obtaining written informed consent from participants. The study focused on patients attending the dermatology OPD during the specified period and diagnosed clinically with PD. Specifically, patients with lesions on any body part, accompanied or unaccompanied by an itching or burning sensation lasting fewer than five days, were included. Patients with lesions attributed to other known causes, such as fungal infections, contact allergies, or atopic dermatitis, were excluded.

The study population was carefully selected through purposive sampling, ensuring all accessible PD patients meeting the inclusion criteria were included using consecutive sampling. Data collection utilized a predefined, semi-structured, pretested questionnaire to gather information on patient demographics, symptom onset, insect contact history, clinical and systemic examination findings, and treatment history. Data were manually entered into Microsoft Excel (Microsoft Corp., Redmond, WA, USA), coded, recoded, and analyzed using SPSS version 23 (IBM Corp., Armonk, NY, USA). Descriptive statistics, including numbers and percentages for categorical variables and mean (with standard deviation, SD) or median (with interquartile range, IQR) for continuous variables, were used for analysis.

## Results

This study included 10 patients presenting to the dermatology OPD with a clinical diagnosis of PD. The mean (SD) age of the patients was 19.4 (1.9) years, ranging between 17 and 22 years. More than half of the patients (60.0%) were males, and 40.0% were females. Of the 10 patients, four (40.0%) were from rural areas living in kutcha houses, three (30.0%) were from urban areas, and three (30.0%) were from semi-urban areas residing in pucca houses. Maximum cases were reported between June and September, the monsoon season. The most common presenting complaint was a burning sensation in 80.0% of the patients, followed by pain in 80.0%, and blisters in 20.0% of the patients.

**Figure 1:**
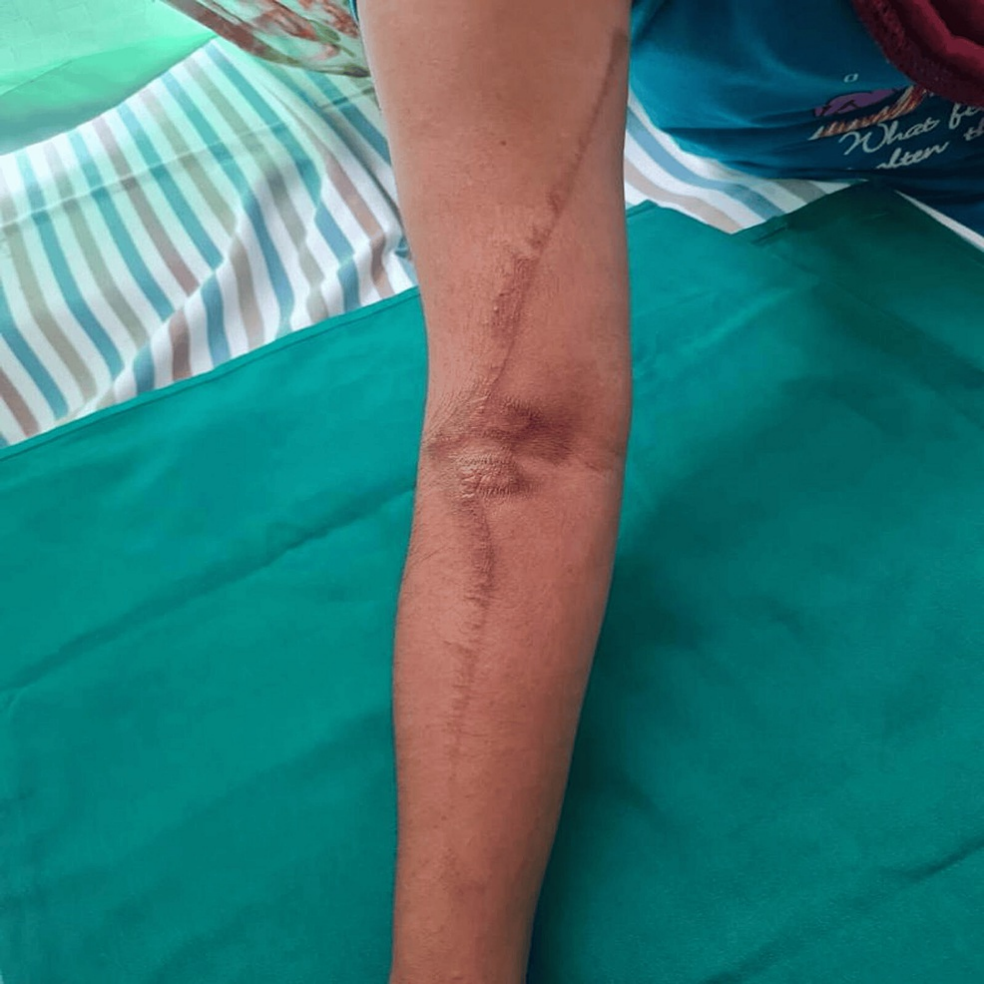
Linear erythematous presentation.

**Figure 2:**
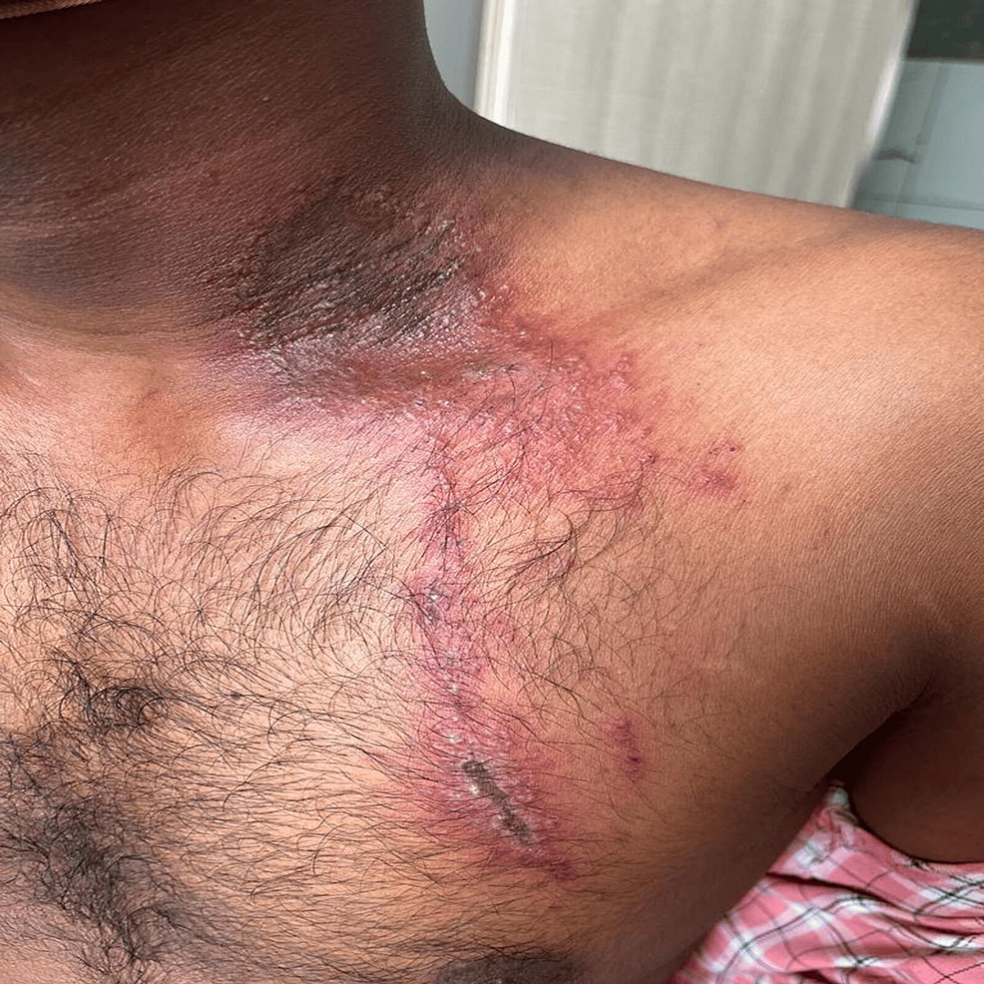
Bizarre erythematous plaques with central necrosis.

**Figure 3:**
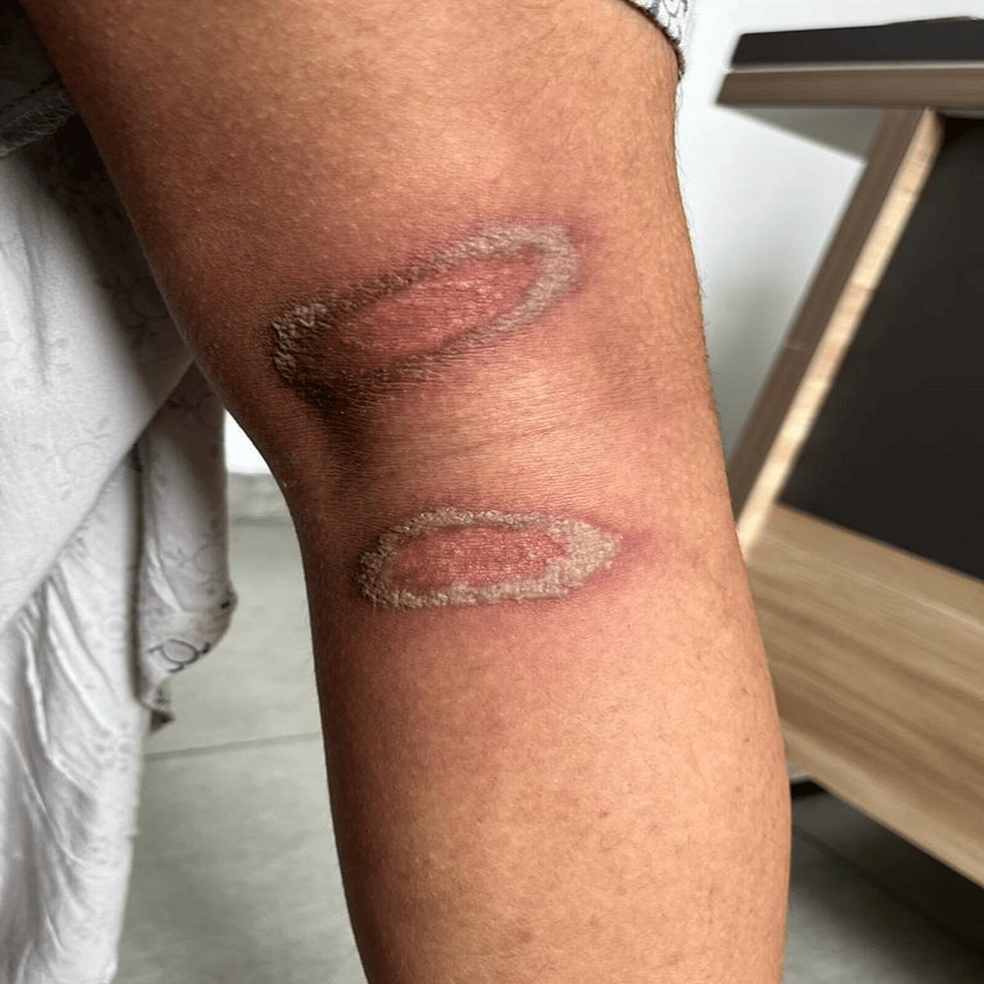
Kissing lesion in paederus dermatitis.

**Figure 4:**
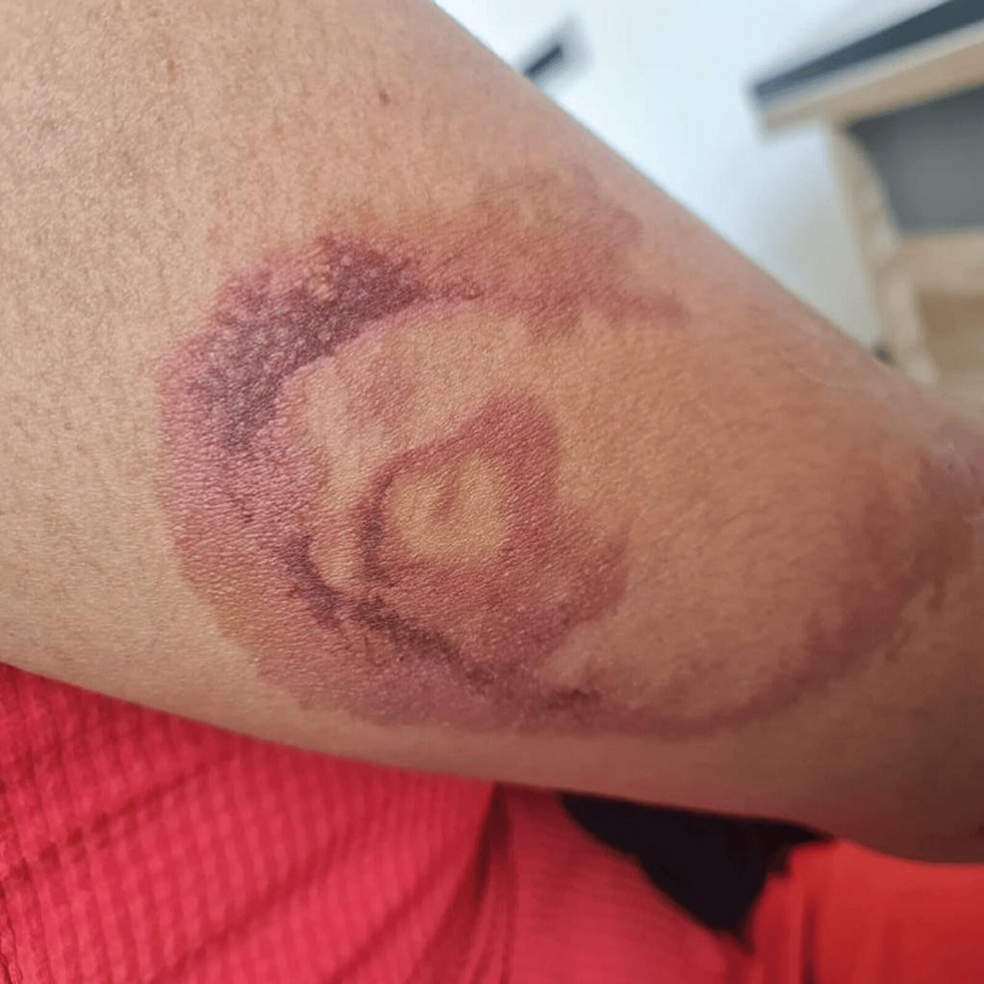
Stellate patch of erythema.

The mean (SD) duration of the lesion was 4.2 (1.3), ranging between two and six days. Although the patients could not recall their encounter with beetles, they all reported experiencing a burning, itchy sensation at the lesion site during the night and noticed fully developed lesions the following morning. Regarding the clinical pattern of the lesions, linear lesions were the most common at 40.0% (Figure 1). This was followed by erythematous lesions with central gray area in 30.0% (Figure 2), kissing lesions (Figure 3) in 20.0%, and burnt appearance in 10.0% (Figure 4) of the lesions.

**Figure 5:**
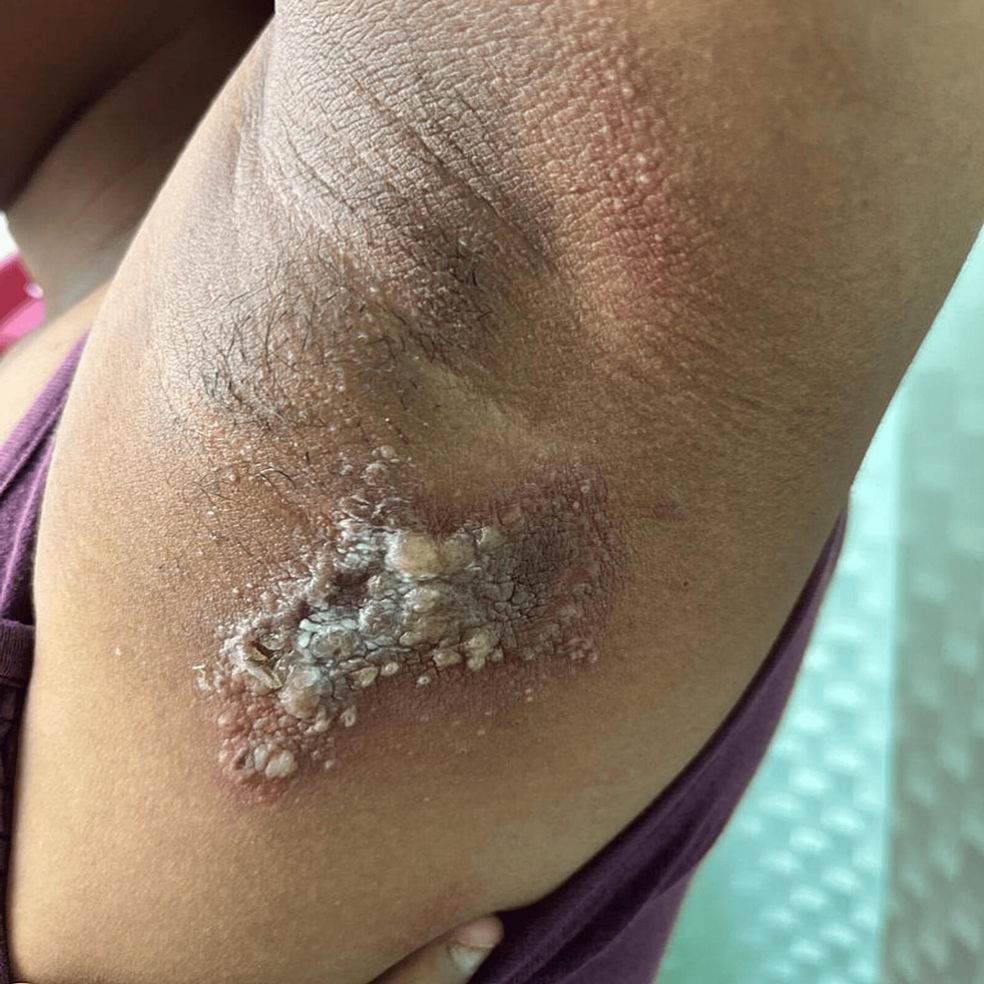
Paederus dermatitis with irritant contact dermatitis.

Regarding the site of the lesions, nearly half of the patients presented with lesions in the face (40.0%), the most common site in the present study, followed by lesions in the leg (20.0%), and lesions in the axilla (Figure 5), chest, arm, and back (10% each).

## Discussion

The findings of this retrospective case series provide valuable insights into the clinical presentation and epidemiology of PD in the study population. PD, commonly known as dermatitis linearis, is an inflammatory skin condition caused by contact with the toxin-containing hemolymph of certain species of beetles belonging to the genus *Paederus*. Beetles induce a type of skin inflammation characterized by papules and vesicles due to an allergic response [7]. The clinical presentation typically involves erythematous lesions with a burning sensation, often occurring in linear patterns, as observed in this study. PD prevails in regions where paddy fields and sugarcane plantations are prominent. Beetles are also drawn to artificial lights, which contributes to their interaction with humans. Instances of dermatitis outbreaks have been documented in various countries, including Australia, Malaysia, Sri Lanka, Nigeria, Kenya, Central Africa, Uganda, Okinawa, Sierra Leone, Argentina, Brazil, France, Venezuela, Ecuador, and India, with established links to *Paederus* beetles [3,4,8-10]. *P. melampus* is a common causative agent of PD in India [11], particularly in the Indian states of Odisha, West Bengal, Punjab, Rajasthan, and Tamil Nadu [7].

The study population comprised predominantly young individuals, with a mean age of 19.4 years, which aligns with previous literature indicating a higher prevalence of PD among younger age groups due to increased outdoor activities and exposure to the insect habitat during certain seasons [12]. The predominance of males (60.0%) in this study is consistent with some previous reports, although the exact reason for this gender predilection remains unclear and warrants further investigation [13]. The distribution of cases across different residential areas provides valuable epidemiological data. The higher proportion of cases from rural areas (40.0%) compared to urban and semi-urban areas suggests a potential association between the habitat of *Paederus* beetles and the incidence of dermatitis. Rural environments, with their proximity to agricultural fields and natural habitats, may offer favorable conditions for beetle breeding and subsequently increase the risk of human exposure to *Paederus* spp. This finding underscores the importance of environmental factors in the epidemiology of PD. The peak time of presentation during June-September corresponds to the monsoon season in the study region, which is consistent with previous studies indicating a seasonal variation in the incidence of PD, with higher rates reported during the warm and humid months [14]. This temporal association can be attributed to the breeding and activity patterns of *Paederus* beetles, which are often more prevalent during the rainy season [15].

The most common presenting complaints of a burning sensation (80.0%) and pain (80.0%) are the hallmark features of PD and are attributed to the vesicant toxin (cantharidin), pederin, present in the hemolymph of *Paederus* beetles. The disease is provoked by an insect belonging to the genus *Paederus*. This beetle does not bite or sting, but accidental brushing against or crushing the beetle over the skin provokes the release of its coelomic fluid, which contains paederin, a potent vesicant agent. The average amount of cantharidin detected was 3.89 μg per beetle in males and 21.68 μg per beetle in females, which are capable of causing skin irritation in humans. Cantharidin dermatitis typically manifests as non-inflammatory vesicles and bullae [16]. These symptoms typically manifest within hours of contact with the insect and are followed by the development of erythematous lesions, as observed in this study [17,18]. The presence of blisters in 20.0% of patients further corroborates the vesicant nature of pederin and its ability to induce blister formation upon contact with the skin. The clinical pattern of lesions observed in this study, including linear lesions (40.0%) and erythematous lesions with a central gray area (30.0%), corresponds to the classical descriptions of PD in the literature [19]. Atypical variants of PD have several possible causes, i.e., (a) contact with different species of *Paederus*; (b) repeated contact within a short period; (c) existence of underlying disorders, e.g., atopic dermatitis; (d) use of heavily infested sources of water for bathing; and (e) immunologic phenomenon resulting in a systematized reaction pattern.

Clinically, PD mimics a host of other dermatoses, e.g., herpes zoster, phytophotodermatitis, impetigo, etc. There are few systemic associations with this condition. Four cases of hospitalization for extensive ulcerations and exfoliative dermatitis were reported by Todd et al [20]. Vasculitis-like eruptions, cervical lymphadenopathy, and nodular interstitial infiltration of the lungs have been reported. Erythema multiforme after contact with beetles has also been reported. Several authors have opined that an important factor in outbreaks of blister beetle dermatitis is proximity to paddy fields [19]. However, we observed that blister beetle dermatitis is a common problem in tropical countries like India. Clinical knowledge about the varied and diverse presentation will help in early diagnosis and treatment. The characteristic linear arrangement of lesions, often referred to as dermatitis linearis, is pathognomonic of PD and results from the repeated linear movements of the insect across the skin during nocturnal activities. The development of classic lesions involves the accumulation of neutrophils within the epidermis along with areas of necrosis and degeneration, whereas lesions characterized by vesicles or pustules result from neutrophilic spongiosis leading to vesicle formation and eventual degeneration of the epidermis [17].

The distribution of lesions, with nearly half of the patients presenting with lesions on the face (40.0%), underscores the importance of nocturnal insect activity and inadvertent contact with *Paederus* beetles during sleep. The proximity of facial lesions to the eyes is of particular concern, as ocular exposure to pederin can lead to severe ocular inflammation and even permanent visual impairment [11].

The present study is not without limitations. The retrospective nature of the study and reliance on patient recall for exposure history may introduce recall bias and limit the accuracy of exposure assessments. Additionally, the small sample size and single-center design may limit the generalizability of findings to other populations. Future studies with larger sample sizes and prospective designs are warranted to further elucidate the epidemiology, clinical features, and management of PD.

## Conclusions

This retrospective case series sheds light on the epidemiology and clinical presentation of PD in the study population. The findings highlight the seasonal variation in the incidence, characteristic clinical features, and distribution of lesions associated with PD. Understanding the epidemiology and clinical manifestations of this condition is crucial for accurate diagnosis, timely management, and public health interventions aimed at preventing *Paederus* beetle-related dermatitis.
